# Mentalization and Parental Stress: How Do They Predict Mother–Child Interactions?

**DOI:** 10.3390/children9020280

**Published:** 2022-02-17

**Authors:** María-Pía Santelices, Pamela A. Cortés

**Affiliations:** Psychology School, Pontificia Universidad Católica de Chile, Vicuña Mackenna 4860, Macul, Región Metropolitana 7810000, Chile; pjcortes@uc.cl

**Keywords:** parent–child interaction, encouragement dimension, teaching dimension, parental stress, mentalization

## Abstract

Parent–child interactions can be negatively influenced by contextual, individual, and familial factors. The present study examines how parental stress and parental mentalization predicts interactions between 36–48-month-old preschoolers and their mothers. The sample comprises 106 mother–child dyads from Santiago, Chile, from a mid-low SES. The instruments used were the Parental Stress Index-Short Form (PSI-SF), Mentalization in the Significant Adult during Interaction with the Child between 10 to 48 months old, and Parenting Interactions with Children: Checklist of Observations Linked to Outcomes (PICCOLO). A regression model was used to determine the significant impacts of mentalization and parental stress on interactions. The results indicate that the Encouragement dimension of parent–child interaction is predicted by emotional mentalization and parental distress, while the Teaching dimension of parent–child interaction is impacted by the cognitive dimension of mentalization and the perception that the child is difficult (stress difficult child). No association for the Responsivity and Affectivity dimension was observed.

## 1. Introduction

The preschool period is characterized by major changes for both the child and his or her parents [1,2]. The management of the transformations associated with this stage of life is greatly influenced by the competencies and qualities of the main caregivers, who must adapt their parenting skills for children to successfully fulfill the tasks of this stage of life. Therefore, the main caregiver plays an especially important role in the child’s developmental process [3]. In this regard, focusing on maternal involvement is essential as 99.6% of children in Chile are being cared for by their biological mothers [4]. Considering this, the present study focused on two mothers’ individual variables that can become risk or protective factors in children’s development. Previous research shows that the presence of such variables predicts mother–child interactions, thus hindering parental competencies [5].

In this study, we chose to work with the Developmental Parenting Model [6], as its instrument allowed us to measure the factors that we believe are essential for our topic. Moreover, we chose this variable as this model has been tested and validated in Chile, which we found to be very relevant as few studies have investigated this topic with the Chilean population. In this model, Parental Interactions are defined as activities that foster the child’s social, cognitive, and linguistic development. Interactions are grouped into four dimensions: Affection, Responsiveness, Encouragement, and Teaching [6]. It is a parenting measure because several specific parental behaviors can vary within these domains [7].

As previously noted, children’s development is affected by interactions with their parents. However, it has been observed that some parental behaviors can be unstable over time because the adult adjusts them according to the child’s age, which might impact parental interactions [8]. McNally, Eisenberg, and Harris [8] suggest that, as a child grows, parents change their ways of interaction by introducing more verbal modes (e.g., reasoning) compared with other forms of discipline, and tend to display less physical affection to encourage autonomy in children. Therefore, this implies new parenting challenges to which caregivers must adapt [8] as the child grows.

Moreover, parental competencies can be positively or negatively affected by individual, social, and contextual factors, such as beliefs about parenthood, perceptions regarding the child, socioeconomic status, and education level, among others [9]. Those individual caregiver variables are relevant when examining the stability or mutability of the set of factors that affect parent–child interactions, based on the assumption that certain parent’s characteristics can alter their children’s development. Parental stress is moderated by different individual variables such as personality, but also by other “external” aspects that do not depend on personal traits (for example, context factors, child’s characteristics, and so on) [10,11].

The literature has shown that the development and learning of children are highly determined by the context in which they grow. An important part of this context is the role of the primary caregiver. This study aimed to analyze the effect of two individual maternal variables that could act as a risk factor for children’s development as they influence the interactions between the mother and child. These are parental stress and mentalization. First, we chose to include parental stress because according to the Longitudinal Survey of Early Childhood (Encuesta Longitudinal de la Primera Infancia, ELPI); 20.3% of mothers/primary caregivers of children in Chile present clinical stress concerning parenthood [12]. On the other hand, we decided to investigate mentalization because this is a key construct in children’s development, which originates from an interpersonal process. Therefore, the capacity of the mother to mentalize the inner states of the child depends greatly on her own capacity to mentalize [13]. We believe this is a fundamental variable to investigate as this will affect the child’s own capacity to mentalize later in life.

In the present study, Parental Stress [14] is defined as a specific type of stress that results from the demands of parenthood, independently of socioeconomic and situational factors [15]. The concept alludes to the negative feelings and internal anguish experienced within the context of parenting [16]. Caregivers might feel that they are unable to cope with their children’s demands, thereby negatively affecting their interaction in the mother–child dyad [17]. Mothers who display high stress levels can feel overwhelmed by their children’s demands, making them less open to respond to their needs [18]. This is especially damaging to their self-perception as mothers [19]. It has also been observed that parents who report higher levels of parental stress tend to have more authoritarian parenting styles, interact more negatively with their children, and are less involved in their growth process [18]. Similar results have been reported by Neece, Green, and Baker [20], who analyzed the transactional relationship between parental stress and children’s behavioral problems in early and middle childhood (ages 3 to 9) using a sample of 237 families. The results suggest that parental stress is a precursor of children’s behavioral problems [20]. Likewise, children’s behavioral problems also cause stress in parents [20]. Thus, high levels of parental stress are directly and significantly associated with rough treatment and aggressive behaviors in children, while rough treatment correlates significantly and directly with parents’ tendency to treat their children roughly, who in turn display more aggressive behaviors [21].

Parental stress levels can vary in response to the demands that emerge as children grow up, shifting from a mother–child interaction based on high dependency and low autonomy (when children are younger) to increased independence and autonomy from parental figures as they grow [22]. Mothers can perceive these new demands as problematic, increasing the level of stress [22].

On the other hand, parental mentalization is another variable that influences child development. The concept of mentalization was introduced by Fonagy, referring to the ability to understand one’s mind and that of others upon the basis of mental states such as intentions, feelings, thoughts, desires, and beliefs [23]. This ability is especially relevant as an adequate level of mentalization facilitates healthy interpersonal relationships, leading to satisfactory and positive social and emotional development [24]. Infants lack a subjective and coherent representation of themselves when they are born; therefore, the development of mentalization in early childhood requires third parties who perceive them as people who think, feel, and do [23]. Parents or caregivers must be able to reflect that experience to the child and respond sensitively to his or her needs, connecting the child’s behavior with his or her mental states, because this will enable the child to develop a mental model of his or her own experience [25]. Thus, parents’ or caregivers’ mentalization plays a fundamental role in children’s development, with Parental Mentalization being defined as parents’ ability to treat their children as psychological agents with their own mental experiences [26,27].

Parental mentalizing functioning can be affected by multiple internal (personal) or external (contextual) factors [28]. Limitations in the mother’s mentalizing functioning can be caused not only by the situations that she is currently experiencing, but also by her ability to represent her own mental states [28]. Thus, parental mentalization plays a key role in helping mothers respond comprehensively to their children’s distress because it enables them to manage negative behavior cycles during moments of child anguish in a better way [29]. Based on the results of a study analyzing the relationship between mothers’ and children’s mentalization and children’s psychopathology in 168 mother–child dyads (children aged 7 to 12 years), it can be deduced that an adequate level of caregiver mentalization is associated with more sensitive and less negative parent–child interactions [30]. In addition, parents with an adequate level of mentalization tend to explain his/her internal world and that of others to the child in terms of mental states, which allows the child to better understand him/herself and others [31].

Parental mentalization and parental stress interact and impact one another. A study by McMahon and Meins (2012) evidenced that mothers who use more mental state words when describing their preschool children reported less parenting stress [32]. The interaction of these two variables impacts the mother–child dyad, because a parent that shows a difficulty when trying to understand her child’s needs could experience lower satisfaction with parenting [32].

Thus, understanding the fundamental role of parental stress and parental mentalization on child development, the main objective of this study was to examine the influence of two individual variables of mothers—parental stress, and parental mentalization– on the interactions observed in the mother–child dyad. It was expected that, the greater the presence of parental stress in mothers of preschool children, the less parental interaction there would be. Moreover, we believed that lower levels of mentalization in mothers of preschool children would be correlated with less parental interaction.

The present study is relevant because the interaction of these factors has not been studied before in Chilean mothers and their children. The results are a contribution to understanding how parental stress affects mentalization and mother–child interactions more in depth. Clinically, this information is relevant as it helps understand the connection between parental stress and the healthy development of children. With this, we can direct our interventions towards parents who show high parental stress, helping them specifically in the areas that we found are more affected by this type of stress. This will help us support their relationship and, therefore, child well-being, as parents’ behaviors and those of everyone who interacts with the child in his/her most immediate context are fundamental because they impact the emergent competencies and the cognitive, linguistic, social, and emotional development of the child [33].

## 2. Materials and Methods

### 2.1. Design

This is a quantitative, non-experimental, and cross-sectional study. The sampling process is non-probabilistic and accidental. 

### 2.2. Participants

The sample comprises 106 mother–child dyads. Regarding the selection process, this study is based on the data collected for Fondecyt Project No. 1130786, “Design, implementation, and evaluation of an Attachment/Mentalization intervention for mothers and fathers of 3-year-old preschoolers”, conducted in preschools belonging to the National Council of Preschools of Santiago, Chile, which mainly receive psychosocially vulnerable families. The sample was collected at seven preschools located in the south of Santiago. Preschoolers and parents participated voluntarily. For inclusion criteria, children must attend preschool regularly and have no special educational needs or diagnosed pathologies. Children were asked to give their assent and their parents agreed to participate in the study by signing an informed consent letter. The children’s age ranged from 10 to 48 months (43.67 months on average), while the mothers’ age ranged from 19 to 47 years (29.59 years on average). The mothers’ education level was diverse: 22% of them had not finished school (studied less than 12th grade), 45% had finished school (studied up to 12th grade), 25% had not completed higher education (started university or higher education degrees, but did not finish), and 6% had completed their higher education studies. 

This sample is characterized by homogeneity, as participants have low or mid-low SES (socioeconomic level). In consequence, the data obtained will not be generalized to other populations given that the sample represents a particular socioeconomic segment.

### 2.3. Instruments

Parenting Interactions with Children: Checklist of Observations Linked to Outcomes (PICCOLO) [6]: The Parenting Interactions with Children: Checklist of Observations Linked to Outcomes (PICCOLO) [6] is an observational instrument that measures positive parenting. It was developed to measure the increase in parental behaviors that support children’s early development. It has 29 items grouped into four dimensions (Affectivity, Responsiveness, Encouragement, and Teaching). It is based on the interactions between parents and children that predict early social development. The affection dimension concerns the physical and verbal expression of affection, positive emotions, positive evaluation, and positive regard [34]. Responsiveness relates to the adult reacting sensitively to the child’s cues and expressions of needs or interests and reacting positively to his/her behavior [34]. Encouragement includes parents’ support of children’s efforts, exploration, independence, play, choices, creativity, and initiative [34]. Finally, teaching relates to cognitive stimulation, explanations, conversation, joint attention, and shared play [34].

The indicators of each item were observed and evaluated in more than 4500 videos of parents with their children aged between 10 and 47 months. They consisted of seven min of free play between the caregiver and the child, being filmed starting at minute two. The instrument provides a subtotal score for each dimension. The maximum score is 14 points in the Affectivity, Responsiveness, and Encouragement dimensions, and 16 points in the Teaching dimension, reaching a maximum score of 68 points. The specific cut-off scores for each dimension are obtained from the study by Roggman et al. [6]. The score is not determined by the presence of specific toys or materials, but rather by what the agent does with the toys or materials and the activities they do with their children. For the present study, two coders who achieved Kappa reliability of 0.79 were required, and the teaching scales (alpha of 0.55), encouragement (alpha of 0.63), affectivity (alpha of 0.63), and responsiveness (alpha of 0.76) were used. 

Parenting Stress Index-Short Form (PSI-SF) [15]: This index provides a measure of stress levels in a short time of administration, assuming that it may be due to situational variables, characteristics of the parents, and/or behavioral characteristics of the children that are related to the role of parenthood. In this way, the instrument is aimed at the parental sphere and is made up of 36 items with a Likert-type response scale. It delivers scores according to three factors or subscales (with 12 items for each one): paternal discomfort, referring to the stress announced by the mother concerning her characteristics associated with the role of motherhood; dysfunctional parent–child interaction, which accounts for the stress perceived by the mother in her interaction with her child; and difficult child, which has to do with how the mother experiences the difficulty or difficulty in controlling her child based on the behavioral traits/characteristics of the child. From the sum of the three subscales, a score is obtained, indicating the level of stress that the mother experiences when exercising motherhood [15]. It has validity and reliability tests, specifically, the short version in Spanish shows an adjustment above 0.95. In this study, the paternal distress subscales were used, with an alpha coefficient of 0.85; the difficult child subscale, with an alpha coefficient of 0.84; and the parent–child dysfunctional interaction subscale, with alpha coefficients of 0.87. 

Instrument for Measuring Mentalization in the Significant Adult during Interaction with the Child between 0 to 48 months old [25]: This instrument was developed with a Chilean sample and aims to analyze verbal interactions between significant caregivers, both family and educational, in interactions with children from 0 to 48 months in a situation in which the adult is asked to tell two stories to their children. The first one is called “Where is mi favorite toy?”. It shows two images, one where a child is playing with a toy in the front yard. The other image shows the same child standing on the door without the toy. The text says “Josephine/Andy is playing with his/her favorite toy in the yard when she’s called to go and have lunch. When she finishes lunch, she remembers she left her toy alone in the yard and goes back to get it…”. The second story also shows two images. In the first one, there is a mother and a child. The mother is carrying a bag and the kid is walking on top of a high sidewalk. In the second image, you can see the kid sitting on the sidewalk and the bag on the floor. The text says: “Marie/Peter is going with her mother back home, after going to buy eggs. He likes to go jumping on the street and the sidewalk. The mother says “Careful, you can fall”. When he jumps, Peter trips, pushing her mother. The instrument uses a structured situation to elicit references to mental and nonmental states in the adult’s speech. Storytelling was chosen because it has already been recognized as the ideal activity for eliciting references to mental states in adult speech during interactions with a child [25]. The coding of the instrument includes evaluating the adult’s speech considering the presence/absence and the number of mentions of six mental contents (desires, cognitions, emotions, attributes, states of consciousness, and physiological states) and support for mental work (causal language, factual language, links with the child’s life, and physical expressions), which allow evaluating the mentalizing capacity of the adult, classifying it as low, adequate, or high. The studies with the instrument report adequate reliability, showing a Cronbach’s alpha coefficient of 0.70 and an average Kappa of 0.72 in the different subscales. For this study, the desires, cognitions, and emotions dimensions were used, whose scores were calculated from the total of words mentioned whose contents alluded to these subscales.

### 2.4. Procedure and Data Analysis

The Scientific Ethics Committee of the institution that develops this research approved this study. After contacting the preschools and explaining the project to them, parents and educational staff were invited to participate voluntarily. The educational staff had a facilitator role during the study. They were the connection between the investigators and families. They coordinated the dates and times of the interviews and observation. Mothers were asked to sign an informed consent letter, while children gave their verbal consent. The evaluation process was conducted in the participating schools. Instruments were administered and coded by raters trained by experts.

The tapes were recorded in an open room in the children’s preschool, next to the playing room. If any discomfort was noticed during the filming of the interaction, the activity was immediately stopped. Children and parents were also informed that they could stop the activity at any given moment. 

The instrument consists of seven minutes of free play time between the parent and child, and the filming starts in minute 2 (therefore, the recording is finally 5 min long). The instruction is for them to play normally like they would do at home on their own. The materials used for playing are standardized and given to them.

The instrument coding consists of evaluating the adult’s speech considering the presence or absence and the number of mentions of desires, cognitions, emotions, attributes, states of consciousness, physiological states, causal language, factual language, links with the child’s life, and physical expressions, which can evaluate the mentalizing capacity of the adult, classifying it as low, adequate, or high.

A group of six psychologists was trained in a course consisting of 20 h and later evaluated until they reached a 0.8 consistency in Kappa coefficient. This training was done by an authorized trainer, who was approved by the instrument authors [6]. When 50% of the tapes were coded, the coders were tested again to ensure that they maintained 0.8 consistency.

Considering that parental interactions constitute the variable of interest in the study, the analyses conducted are focused on identifying what variables can predict those interactions. To do this, a *Pearson correlations matrix* was generated with the variables studied to establish whether any associations exist among the variables of interest. Finally, a *multiple regression analysis* was performed to determine whether the predictor variables (parental mentalization and parental stress) are significant predictors of parental interactions, when controlling for other variables, to get a clearer picture of each variable as an explanatory factor of parent–child interactions.

## 3. Results

In this section, the descriptive statistics that characterize the population in the variables of interest for this study are presented. In general, the levels of parental stress in the three dimensions that were used in this study (difficult child, parental distress, and dysfunctional interaction) were within the values close to the average of the scale (score 3), considering that the instrument consists of a Likert scale of 1 to 5 (see Table 1). This scale does not have cut-off scores distinguishing by dimension, so it is not possible to establish the presence/absence of clinical stress levels for each dimension.

Table 2 shows the correlation matrix for the variables studied. Regarding the hypothesis that parental stress correlates negatively with parental interactions, a significant negative correlation was observed between parental distress, which refers to the stress associated with exercising one’s parental role [15], and the Encouragement dimension (r(90) = −0.26, *p* = 0.012). This indicates that, the more distress the mother experiences when engaging in her “maternal role”, the lower the presence of interactions in which she encourages and motivates her child.

In addition, a marginally significant correlation was found between the difficult child subscale, which refers to parents’ perception of how easy or hard it is for them to control their children depending on their behavioral traits [15], and the Teaching dimension (r(90) = −0.20, *p* = 0.058). This revealed that mothers who find it more difficult to control their children’s behavior engage in fewer interactions aimed at giving explanations and playing in a way that fosters their child’s learning and autonomy. 

However, no significant correlations were found between any of the parental stress dimensions and the Responsiveness dimension, nor between any of the parental stress dimensions and the Affection dimension.

These results partially confirm the initial hypothesis that more parental stress tends to be associated with lower parent–child interaction levels. The confirmation is only partial because not all the parental stress dimensions displayed an association with the multiple dimensions of parent–child interactions.

Regarding the hypothesis that the mother’s mentalizing ability (parental mentalization) is positively associated with the dimensions included in parental interactions, we observed that the desires mental content, that is, words or phrases that refer to wishes, preferences, and intentions [25], correlates positively with the Encouragement dimension (F(90) = 0.28, *p* = 0.007). This indicates that mothers who use more words that allude to intentions and motivations are more likely to encourage and motivate their children in their interactions. Likewise, the emotion mental content, which occurs when the adult explicitly refers to feelings, emotions, or moods [25], correlates positively with the Encouragement dimension (r(90) = 0.30, *p* = 0.004); therefore, mothers who use words that describe emotions and feelings tend to encourage and motivate their children more often during their interactions. Thus, high scores of encouragement interactions are associated with high scores of emotion mentalization.

Finally, it was observed that the mental content cognition, which refers to the adult’s use of words referred to mental/cognitive processes [25], correlates positively with the Teaching dimension (r(90) = 0.39, *p* < 0.001). This indicates that mothers who use a type of language that alludes to mental states such as reasoning, understanding, and remembering tend to provide their children with more explanations and teachings in their interactions. Therefore, high scores of teaching interactions are associated with high scores of cognition mentalization. Those findings partly confirm the hypothesis that mentalization correlates positively with parental interactions. Significant correlations were only found between the desires and emotions mental contents and the Encouragement dimension, while the cognition mental content correlated positively with the Teaching dimension of parental interactions.

Only the Teaching and Encouragement dimensions (parental interactions) were found to display significant associations with the predictor variables, that is, with subcategories associated with parental stress and with mental contents linked to mothers’ mentalizing capacity. However, merely analyzing these correlations does not provide enough evidence to assert that the explanatory variables of the study affect parents’ parental interactions. To fulfill this task, multiple regression analyses will be conducted including the explanatory variables that displayed significant correlations with the dependent variables. Given the lack of significant correlations, it was decided not to employ multiple regression models with the indicators from the Affection and Responsiveness dimensions in parental interactions.

To generate the regression models, child sex, child age, and mother’s age were incorporated as covariates. The mother’s education level was also added given that it is a relevant variable in this study. This approach is aimed at controlling for the factors not considered in the study design that could affect the relationship between the dependent variable and the explanatory variables, in order to obtain estimators that reflect the unique effect of each variable. Table 3 presents the results of the Encouragement dimension regressed on the predictor variables included in the study. The results show that the emotion mental content and the parental distress subscale have significant effects, even when controlling for the whole set of variables included in the model. Moreover, the model as a whole was found to be significant, predicting 26% of the variance of the Encouragement dimension.

Table 4 presents the results of the Teaching dimension regressed on the predictor variables included in the study. The results show that the cognition mental content and the parental stress subcategory difficult child have significant effects, even when controlling for the whole set of variables included in the model. Moreover, the model as a whole was found to be significant, predicting 35% of the variance of the Teaching dimension.

## 4. Discussion

The present quantitative study sought to analyze the effects of parental stress and parental mentalization on parent–child interactions in preschoolers’ mothers.

Regarding the predictor variable parental stress and its repercussions on parental interactions, we observed that the Encouragement dimension (parental interactions) is predicted by the paternal distress subcategory (parental stress) in the regression model. These results are consistent with reports that mothers with higher levels of parental stress tend to have more authoritarian parenting styles, interact more negatively with their children, and are less involved in their growth process, all of which make it less likely for mothers to actively support their children’s exploration, effort, skills, initiative, curiosity, and play [7,18].

The second regression model showed that the Teaching dimension (parental interactions) is significantly predicted by the difficult child subcategory (parental stress). This dimension of parental stress refers to parents’ perception of how easy or how hard it is for them to control their children given the latter’s behavioral traits [15]. This association, which was found to be negative, is in line with studies that show that parental stress is both a precursor and a consequence of child behavior problems [20]. For instance, Cabrera, Gonzales, and Guevara [21] report that high levels of parental stress are associated with interactions based on a rough treatment from parents, which result in aggressive behaviors from children toward their parents. Based on this evidence, it could be hypothesized that high levels of parental stress reduce the presence of parental behaviors included in the Teaching dimension such as cognitive stimulation, explanations, conversation, joint attention, and shared play [7].

The parental mentalization variable was analyzed through three specific mental states: desires, cognitions, and emotions. The results obtained show that these mental contents (desires, cognition, and emotions) have a positive effect on the Encouragement and Teaching dimensions of parental interactions.

Finally, although it was not considered—from the beginning of the study—to analyze the effect of the mother’s level of education, we decided to carry out an exploratory analysis using simple ANOVA, as this factor turned out to be marginally significant in the regression model. Therefore, it was decided to delve into the differences between the different groups of education in mothers concerning parental interactions. In this regard, post hoc analyses showed that mothers with complete higher education have significantly higher averages than mothers with incomplete or complete schooling in the Teaching dimension only. This is consistent with all the literature that refers to the explanatory value of the educational level in parenting, especially in aspects related to learning and exploration [35]. A higher educational level in the mother could increase parental self-efficacy around understanding the world, which would allow a greater availability of the mother to communicate with the child by talking and answering their questions, trusting that she knows the answer, which—in the same way—promotes an affective bond [36]. In the same way, those parents who have a lower educational level would have, at least, more difficulties to correctly interpret the behaviors and attitudes of children, and thus respond appropriately to their needs reflected through mother–child interactions [37]. The foregoing is explained by understanding that a better level of instruction implies greater access to information; the acquisition of knowledge, skills, values, and attitudes required to be able to generate responses for the care and education of children; promoting their healthy development; and the deployment of its potentialities [37].

Our hypothesis was partially confirmed because we observed a negative and significant correlation between parental distress and the Encouragement dimension. Moreover, a marginally significant correlation was observed between the difficult child subscale and the Teaching dimension. Nevertheless, we found no other significant correlations between any of the parental stress dimensions and the Responsiveness dimension, nor between any of the parental stress dimensions and the Affection dimension. 

Thus, based on the analysis of the results of the regression model employed, it can be stated that the cognition mental state (in which the adult uses words related to mental/cognitive processes) has a positive effect on the Teaching dimension of parental interactions. The same is true of the mental contents emotions (referring to feelings, emotions, or moods) and desires (words or phrases that refer to wishes, preferences, and intentions), which correlate positively with the Encouragement dimension of parental interactions. These results are consistent with evidence showing that parental mentalization plays a key role in helping mothers respond comprehensively to their children’s distress because it enables them to better manage negative behavior cycles during moments of child anguish [29]. Therefore, the authors suggest that adequate parental mentalization is associated with more sensitive and less negative parental interactions, which allows parents to handle vulnerability in children without interference from children’s emotions [30]. This situation can be understood considering that these mothers tend to explain his/her internal world and that of others to the child in terms of mental states, which allows the child to better understand him/herself and others [31].

In this study, accepting that the dimensions of parental interactions are separable also entails the need to search for, or develop, theories that shed light on why certain explanatory variables are only linked to specific dimensions of the parental interaction construct but not to others (such as Affection and Responsiveness). This situation was observed in the present study, as significant associations were found only for the Teaching and Encouragement dimensions, not for the Affection and Responsiveness dimensions. Given this finding, it is necessary to continue researching this issue. The results of this research can be used in the context of clinical psychology, allowing the identification of the effects of certain parental variables on parental interactions, which can provide a relevant background to create interventions or actions that support the process of parenthood.

The results of this study are a contribution to understanding more in-depth how parental stress affects mentalization and mother–child interactions. Clinically, this information is relevant as it helps understand the connection between parental stress and the healthy development of children. With this, we can direct our interventions towards parents who show high parental stress, helping them specifically in the areas we found that are more affected by this type of stress. This will help us support their relationship and, therefore, child well-being.

Concerning the limitations of the present study, it should be noted that the sample used was socioeconomically and educationally homogeneous (mid-low SES). The size of the sample (106 mother–child dyads) was also restrictive, and it is not fully representative of Chilean society because it only studied families that lived in Santiago and children that attended nurseries. This socio-demographic variable is a very important aspect, which—although it was not considered within the objectives of this study—is a key element to consider in future research to explain the development of children and could, in turn, be considered a predictor and protective variable of their development [37]. In addition, it must be pointed out that the size of the sample could have reduced the study’s statistical power, making it less capable of identifying significant associations present in the population. Thus, it is necessary to replicate these results with larger and more heterogeneous samples in future research. Likewise, future studies could include father–child dyads to further enrich scientific approaches to parental interactions. Finally, it is necessary to point out that some values of Cronbach alpha’s are at the edge of acceptability. This has been observed in various studies using attachment observational instruments, but information should be used carefully. 

Finally, another aspect to be considered is the fact that the tapes are recorded in a “non-natural” environment, which can distort the mother–child relationship, making it different from usual. Therefore, it is important to consider the effect of social desirability, which can be greater as parents know that their interactions are being recorded.

## Figures and Tables

**Table 1:** Descriptive statistics.

	Mean	Observed Range	Scalar Range
Difficult Child	2.13 (0.68)	1.08–4.08	1–5
Parental Distress	2.25 (0.76)	1.00–4.58	1–5
Dysfunctional Interaction	1.58 (0.60)	1.00–4.25	1–5
Mentalization: Desires	1.10 (1.35)	0–7	0–∞
Mentalization: Cognition	1.32 (1.68)	0–6	0–∞
Mentalization: Emotion	0.82 (1.35)	0–6	0–∞
Interaction: Teaching	7.90 (2.14)	2–12	0–16
Interaction: Encouragement	8.95 (2.41)	3–14	0–14
Interaction: Responsiveness	11.52 (2.44)	5–14	0–14
Interaction: Affection	6.84 (1.37)	3–8	0–14

**Table 2:** Correlation matrix.

	1	2	3	4	5	6	7	8	9
1. Difficult child									
2. Parental distress	0.52 ***								
3. Dysfunctional Int.	0.53 ***	0.51 ***							
4. Ment.: desires	−10.3	0.10	−0.17						
5. Ment.: cognition	0.12	0.18	−0.09	0.17					
6. Ment.: emotion	−0.12	−0.01	0.00	0.22 *	0.10				
7. Int.: Teaching	−0.21 *	−0.02	−0.18	0.04	0.39 ***	0.18			
8. Int.: Encouragement	−0.13	−20.6 *	−0.12	0.28 **	0.03	0.30 ***	0.38 ***		
9. Int.: Responsiveness	−0.11	−0.16	−0.13	0.09	0.13	0.07	0.34 ***	0.54 ***	
10. Int.: Affection	−0.11	−0.00	−0.08	−0.04	0.07	0.11	0.44 ***	0.37 ***	0.51 ***

* *p* < 0.05, ** *p* < 0.01, *** *p* < 0.001.

**Table 3:** Encouragement dimension regressed on predictor variables.

	β	t	Significance
Intercept	10.582	9.69	<0.001
Sex (Children)	−0.070	0.04	0.972
Children’s age (months)	−0.057	−0.66	0.513
Mothers’ age (years)	0.000	0.01	0.990
Full school education	0.404	0.60	0.551
Incomplete higher education	−0.366	−0.49	0.622
Full higher education	1.228	1.06	0.292
Mentalization: Desires	0.333	1.62	0.111
Mentalization: Emotion	0.392	2.08	0.041
Stress: Parental Distress	−1.005	2.48	0.015
Stress: Difficult Child	−0.044	−0.10	0.920
Model	R^2^ = 0.26	F(12,69) = 2.01	0.03

Ages are centered on the average value. Incomplete school education was used as a point of reference.

**Table 4:** Teaching dimension regressed on predictor variables.

	β	t	Significance
Intercept	9.253	9.76	<0.001
Sex (Children)	−0.074	−0.16	0.869
Children’s age (months)	−0.033	−0.43	0.666
Mothers’ age (years)	0.049	1.50	0.137
Full school education	0.830	1.42	0.161
Incomplete higher education	0.855	1.33	0.187
Full higher education	1.870	1.86	0.066
Stress: Parental Distress	−0.069	−0.20	0.843
Stress: Difficult Child	−1.052	−2.73	0.007
Model	R^2^ = 0.35	F(12,69) = 3.14	0.001
R^2^ adjusted = 0.24			

Ages are centered on the average value. Incomplete school education was used as a point of reference.

## Data Availability

The data of this study are not available in relation to the confidentiality and the use of the data determinations of one of the financing institutions.
